# Supplementary material for: Effects of sn-2 Palmitic Triacylglycerols and the Ratio of OPL to OPO in Human Milk Fat Substitute on Metabolic Regulation in Sprague-Dawley Rats
Source: Nutrients. 2024 Apr 26;16(9):1299. doi: 10.3390/nu16091299 (PMC11085268; doi:10.3390/nu16091299)
Supplement: Supplementary file 1 [file nutrients-16-01299-s001.zip › nutrients-2951049-supplementary.pdf]

Effects of Sn-2 Palmitic Triacylglycerols and the Ratio of OPL to OPO in Human Milk Fat Substitute on Metabolic Regulation in Sprague-Dawley Rats

Lin Zhu [1], Shuaizhen Fang [1], Yaqiong Zhang [1,] * Xiangjun Sun [1], Puyu Yang [1], Weiying Lu[1], and Liangli Yu [2]

[1] Institute of Food and Nutraceutical Science, School of Agriculture and Biology, Shanghai Jiao Tong University, Shanghai 200240, China

[2] Department of Nutrition and Food Science, University of Maryland, College Park, Maryland 20742, United States

* Corresponding Author:

Yaqiong Zhang, Ph.D. Tel: (86)-21-34204538; Fax: (86)-21-34204538; E-mail: yqzhang2006@sjtu.edu.cn

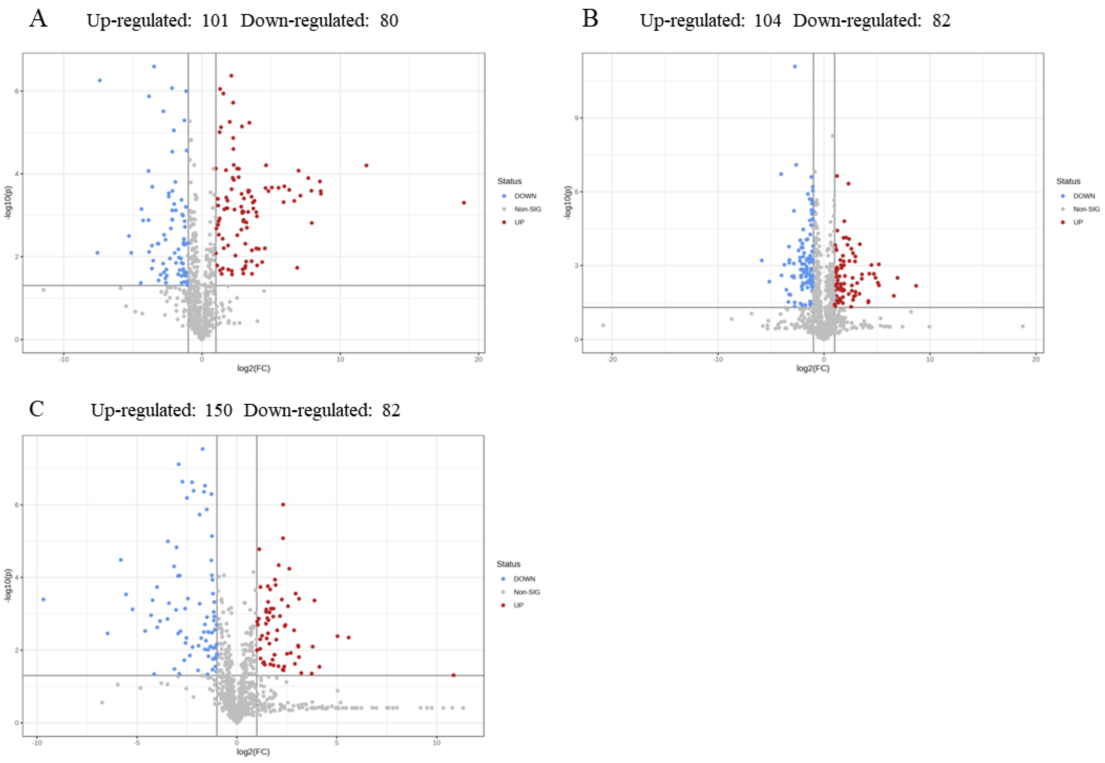

Figure S1. Volcano plot analysis of serum metabolites between CF and HMFS1-fed rats (A), HMFS1 and HMFS2-fed rats (B), HMFS1 and HMFS3-fed rats (C).

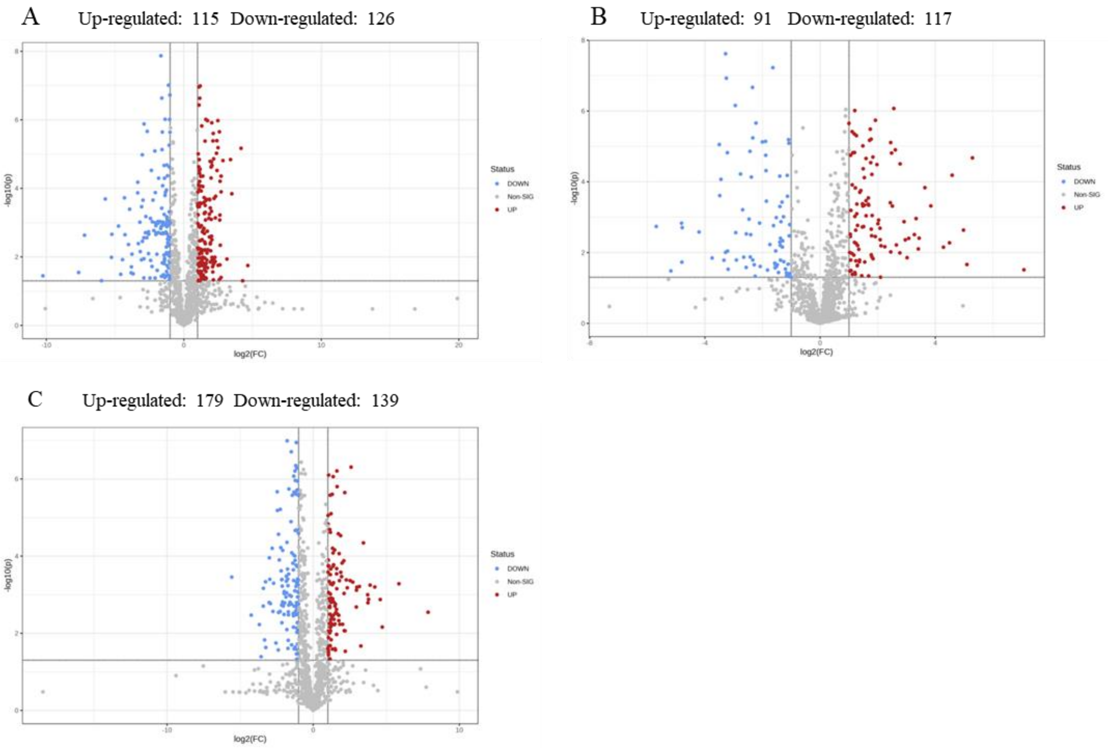

Figure S2. Volcano plot analysis of serum lipids between CF and HMFS1-fed rats (A), HMFS1 and HMFS2-fed rats (B), and HMFS1 and HMFS3-fed rats (C).

Table S1. Fatty acid composition, *sn*-2 palmitic acid content and OPL to OPO ratio in four experimental fats.

	CF	HMFS1	HMFS2	HMFS3
C8:0 (%)	0.52	0.32	0.41	0.28
C10:0 (%)	0.62	0.63	0.63	0.66
C12:0 (%)	3.45	1.68	2.38	2.14
C14:0 (%)	3.09	3.23	3.18	3.39
C16:0 (%)	21.78	22.12	24.08	23.42
C16:1 (%)	0.26	0.37	0.32	0.35
C18:0 (%)	4.70	4.57	4.67	4.82
C18:1 (%)	41.37	42.06	39.45	39.10
C18:2 n-6 (%)	19.23	20.36	19.75	20.81
C18:3 n-3 (%)	3.00	2.05	2.51	2.58
Sn-2 palmitic acid content (%)	15.54	54.36	60.02	57.87
OPL to OPO ratio	0.4	0.3	0.9	1.4

Table S2. The detailed diet formulations.

	CF		HMFS1		HMFS2		HMFS3	
	gm%	kcal%	gm%	kcal%	gm%	kcal%	gm%	kcal%
Protein	22.2	20.3	22.2	20.3	22.2	20.3	22.2	20.3
Carbohydrate	53.3	48.8	53.3	48.8	53.3	48.8	53.3	48.8
Fat	15.0	30.8	15.0	30.8	15.0	30.8	15.0	30.8
Total		100		100		100		100
Ingredient	gm	kcal	gm	kcal	gm	kcal	gm	kcal
Casein	200	800	200	800	200	800	200	800
L-Cystine	3	12	3	12	3	12	3	12
Corn Starch	255	1020	255	1020	255	1020	255	1020
Maltodextrin 10	132	528	132	528	132	528	132	528
Sucrose	91	364	91	364	91	364	91	364
Celluse, BW200	50	0	50	0	50	0	50	0
Fat Sample CF	137	1233						
Fat Sample HMFS1			137	1233				
Fat Sample HMFS2					137	1233		
Fat Sample HMFS3							137	1233
t-Butylhydroquinone	0.0274	0	0.0274	0	0.0274	0	0.0274	0
Mineral	35	0	35	0	35	0	35	0
Vitamin	10	40	10	40	10	40	10	40
Choline Bitartrate	2.5	0	2.5	0	2.5	0	2.5	0
